# Fulminant Leptospirosis Presenting with Rapidly Developing Acute Renal Failure and Multiorgan Failure

**DOI:** 10.3390/biomedicines12020435

**Published:** 2024-02-15

**Authors:** Yu-Hsien Liu, Yu-Hsuan Chen, Chuan-Mu Chen

**Affiliations:** 1 Department of Life Sciences, Doctorial Program in Translational Medicine, National Chung Hsing University, Taichung 402, Taiwan; yuhsien000@yahoo.com.tw (Y.-H.L.); yhchen1218@smail.nchu.edu.tw (Y.-H.C.); 2 Department of Internal Medicine, Jen-Ai Hospital, Dali Branch, Jen-Ai Medical Foundation and Chang Gung Medical Foundation Cooperation Alliance, Taichung 402, Taiwan; 3 The iEGG and Animal Biotechnology Research Center, The Rong Hsing Research Center for Translational Medicine, National Chung Hsing University, Taichung 402, Taiwan

**Keywords:** leptospirosis, jaundice, acute renal failure, Weil’s disease, hemodialysis

## Abstract

Leptospirosis, caused by pathogenic spirochetes of the *Leptospira genus*, is a common zoonosis in tropical and subtropical regions and can lead to an epidemic following heavy rainfall or flooding. The primary reservoirs of *Leptospira* include rodents, wild animals, dogs, cats, amphibians, and others, but the brown rat (*Rattus norvegicus*) remains the main source of human Leptospirosis. Humans are often accidental hosts and they can be infected through cuts, abrasions, mucosa, conjunctiva, or by ingesting contaminated water. The clinical manifestation of leptospirosis can vary from mild, nonspecific symptoms to a fatal outcome involving liver and renal failure, pulmonary hemorrhage, meningitis, and septic shock. The severity of fatal outcomes is likely to be due to virulence factors, host susceptibility, and epidemiological conditions. *L. interrogans* are associated with high-risk individuals, particularly patients older than 60 years of age in clinical settings. The current case study showed a foreign worker who presented with rapidly deteriorating clinical signs of fever, jaundice, impaired consciousness, and oliguric acute renal failure. Drawing from our experience, it is advisable to consider the possibility of leptospirosis diagnosis in patients who show clinical symptoms such as fever, hepatic failure with jaundice, and acute renal failure. This is particularly important for those individuals with a prior history of pathogen exposure. This case study had a strong suspicion of leptospirosis, which was confirmed by the microscopic agglutination test (MAT) and, later, the patient’s recovery following treatment.

## 1. Introduction

Leptospires are thin, spiral-shaped, and motile organisms with endoflagella. They belong to a group of bacteria that includes at least 12 pathogenic and 4 saprophytic species. There are more than 250 pathogenic serovars that can cause systemic infections in both humans and domestic/wild animals, leading to symptoms such as fever, kidney, and liver failure, lung problems, and reproductive failure. Leptospires are obligate aerobes, which means that they require oxygen to survive, and they grow optimally at temperatures between 28 and 30 °C [1]. The most common serotype is *L*. *icterohaemorrhagiae*, typically found in rats (*Rattus norvegicus*). The severity of clinical symptoms can vary significantly depending on the infecting serovar of *Leptospira*, the patient’s age, fitness level, and immune response. It can range from a mild febrile illness to a severe life-threatening condition with multiple-organ involvement, classically known as Weil’s disease [2,3].

Leptospirosis is a global and potentially lethal zoonotic disease that can lead to outbreaks in many tropical and subtropical regions following heavy precipitation and flooding. Recently, it has garnered global attention as a resurging infectious disease caused by a pathogenic spirochete. Infection occurs through direct or indirect contact with the urine of infected reservoir hosts, such as rats, wild animals, and domestic animals. Pathogenic *Leptospira* infect humans through the human body via skin abrasions, cuts, or mucous membranes. Although most patients present mild, nonspecific febrile symptoms, some may experience a fatal clinical course with multiorgan involvement, which is commonly referred to as Weil’s disease [2,3]. The severity of fatal outcomes is likely due to virulence factors, host susceptibility, and epidemiological conditions [3]. The risk of mortality increases with age, particularly in patients over 60 years old. Compared to individuals aged 19–29 years, the risk of death for those aged 40–49 years is 3.7 times higher, and for those aged 60 or older, it is 7.3 times higher [4]. Severe leptospirosis may trigger a cytokine storm, characterized by elevated serum levels of IL-6, IL-10, and TNF-alpha [3], indicating widespread dissemination of the pathogen.

Leptospirosis typically presents as an undifferentiated, acute febrile illness with symptoms like fever, fatigue, myalgia, and headache. It may be mistaken for other diseases, such as influenza, hantavirus infection, or dengue fever [5]. Timely and accurate diagnostic methods are essential for early diagnosis and effective antibiotic treatment.

The kidney is the primary target in leptospirosis, with clinical presentations ranging from abnormal urinary sediment to acute renal failure requiring renal replacement therapy. The incidence of acute renal failure is about 40 to 60% in severe leptospirosis. Hypotension-induced hemodynamic changes, immune responses, and direct nephrotoxicity all contributed to the development of acute kidney injury. Tubulointerstitial nephritis was also a common finding with clinical tubular dysfunction [6]. Acute kidney injury may manifest with non-oliguric or oliguric patterns. When oliguric acute renal failure occurs, prompt initiation of renal replacement therapy can be life-saving. Hepatocellular damage and disruption of intercellular junctions between hepatocytes can lead to bilirubin leakage from bile canaliculi, resulting in hyperbilirubinemia [3]. Coagulation abnormalities due to liver damage may also be closely associated with hemorrhagic complications. The incidence of pulmonary involvement in leptospirosis ranges from 20% to 70%, characterized by dyspnea, hemoptysis, and radiographic findings that show diffuse small opacities disseminated or coalescing into larger areas of consolidation [7].

The patient resides in an urban area and the contraction of leptospirosis was attributed to the consumption of contaminated water. This study presents a case, who had a rapidly deteriorating clinical condition, characterized by oliguric acute renal failure, jaundice, cardiovascular collapse, and subsequent involvement of the lungs and brain. The patient exhibited a severe form of leptospirosis (Weil’s disease) with symptoms such as jaundice, acute renal failure, cardiovascular collapse, and subsequent involvement of the lungs and brain. Even in urban areas, healthcare providers should consider the possibility of leptospirosis when patients present with acute febrile illness, jaundice, and acute renal failure.

## 2. Materials and Methods

A thirty-eight-year-old man who had no previous medical history was admitted to the nephrology department due to fever and acute renal failure. The patient’s medical examination indicated that he had experienced slight nausea and stomach upset, progressive bilateral calf pain, and headaches for the past 3 days, followed by a sudden fever. He also noticed his urine changing to an orange-hued color and a decrease in urination. The patient did not take any medications prescribed by the outpatient clinic. He is a foreign worker from Thailand and has been working in a plastic factory in Taiwan for the last 3 years.

He lived in a staff dormitory located on the outskirts of an urban setting. There are some stray animals around that area. In his daily life, most of the domestic water is taken from groundwater. He has not gone on any recent tours and denied being bitten by any animals. The patient also denied experiencing diarrhea, cough, shortness of breath, skin rash, or any contact with sick individuals. Moreover, he refrains from smoking and abstains from alcohol consumption.

**Figure 1:**
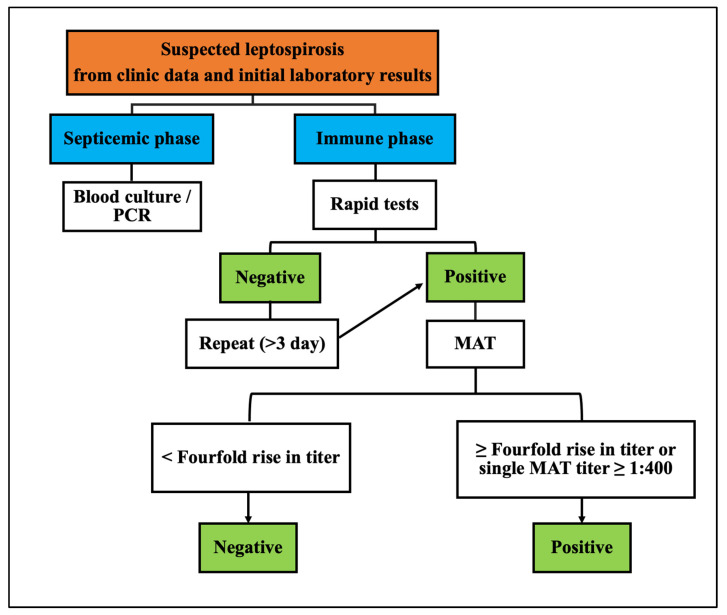
Diagnosis algorithm for leptospirosis.

Upon admission to the department, the patient was conscious and oriented but appeared acutely unwell. Physical examination revealed an elevated body temperature (up to 39 °C), a rapid pulse rate of 142 beats/min, and a normal blood pressure of 102/72 mmHg. Additionally, the patient exhibited slightly dry and yellow skin, mild conjunctival suffusion of the whites of the eyes, and local tenderness over both thighs and calf muscles. The auscultation of the lungs and heart was normal, except for a regular rapid heartbeat. The abdomen was soft with normal peristalsis. The liver was palpable under the right costal arch, whereas the spleen was not palpable. Peripheral pulsations were detectable and no signs of meningeal irritation were found. A clinical data provision consent form for publication has been signed by this individual patient. A diagnosis algorithm for leptospirosis is shown in Figure 1. The diagnosis of leptospirosis was confirmed through the microscopic agglutination test (MAT), conducted by the Centers for Disease Control and Prevention (CDC), Atlanta, GA, USA. The MAT, considered the gold standard for serodiagnosis of leptospirosis, utilizes live leptospires mixed in a dilution series with the patient’s serum. Diagnosis was based on seroconversion or a ≥4-fold titer rise in paired sera [8].

## 3. Results

### 3.1. Clinical Presentation and Initial Treatment Response of Leptospirosis

**Figure 2:**
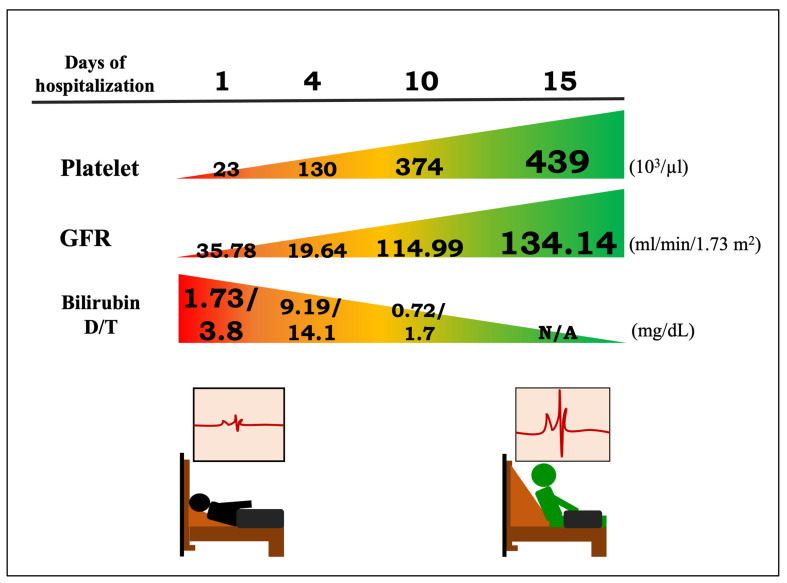
The improvements in platelet count in the blood, glomerular filtration rate (GFR), and serum bilirubin levels during the course of hospitalization.

**Figure 3:**
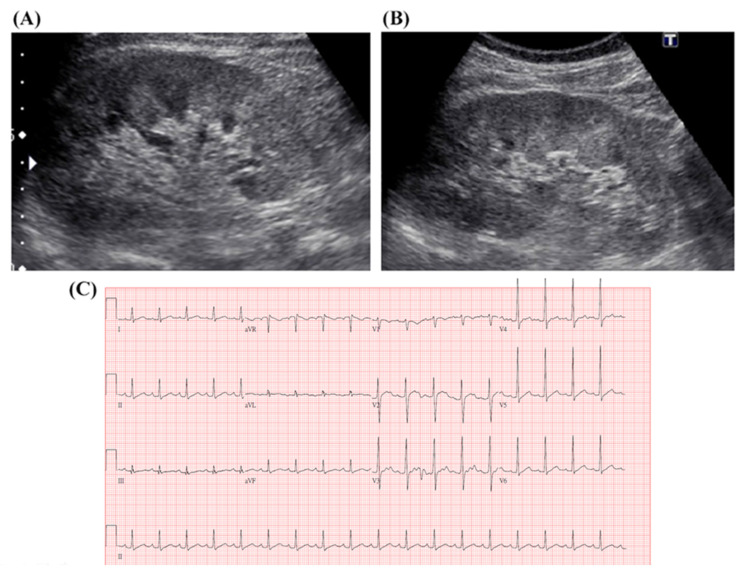
Abdominal ultrasound images and electrocardiography of the patient. (**A**) Right kidney size: 11.07 cm. (**B**) Left kidney size: 12.56 cm. The abdominal ultrasound showed mild enlargement of the left kidney and increased echogenicity in both kidneys. (**C**) The electrocardiography revealed sinus tachycardia with a heart rate of 108 beats/min.

The laboratory test results and vital sign changes during hospitalization (Table 1) were remarkable for an elevated renal profile: creatinine 2.2 mg/dL (normal range: 0.7~1.3 mg/dL), urea 25 mg/dL (normal range: 7~25 mg/dL), glomerular filtration rate (GFR) 35.78 mL/min/1.73 m^2^, and high serum bilirubin with an initially normal liver function indicator (alanine aminotransferase (ALT) of 39 U/L), direct bilirubin (D) of 1.73 mg/dL, and total bilirubin (T) of 3.8 mg/dL, reaching the highest values (bilirubin D/T: 9.19 mg/dL vs. 14.1 mg/dL) on the fourth day of hospitalization. The complete blood count showed thrombocytopenia with platelets 23,000 cells/mm^3^, anemia with hemoglobin 10.8 g/dL, and leukocytosis with white blood cell count 13.76 × 10^3^ cells/mm^3^ (Figure 2). General urine analysis showed moderate proteinuria (2+), hematuria (RBC: 8–15/HPF), and pyuria (WBC: 16–30/HPF). The chest radiological findings were normal. Electrocardiography showed sinus tachycardia rhythm at 142 beats/min. Abdominal ultrasound examination revealed slightly enlarged kidneys with increased cortical echogenicity (Figure 3). The patient was admitted to the general ward with the initial impression of sepsis and acute kidney injury after the initial symptoms for the past 4 day. 

### 3.2. Intensive Care Unit Admission and Evolving Treatment Strategies of Leptospirosis

**Figure 4:**
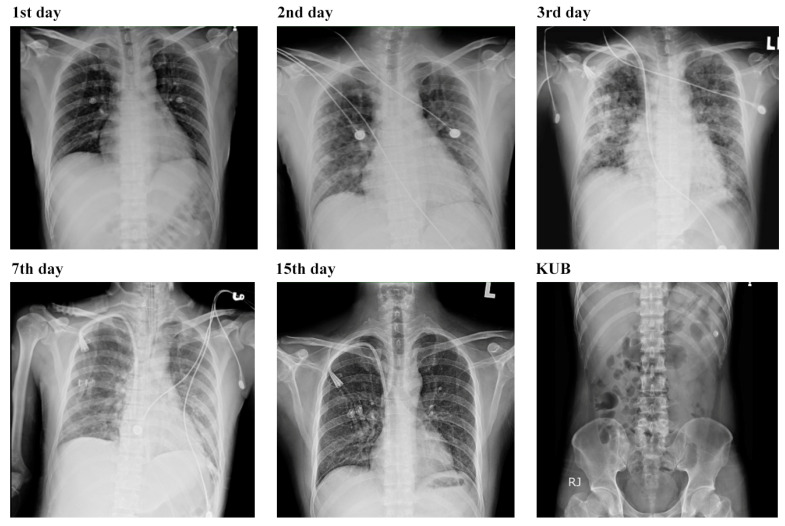
Sequential changes in chest and KUB (kidney, ureter, and bladder) X-rays throughout the hospitalization. The serial chest films, from left to right, show the 1st, 2nd, 3rd, 7th, and 15th days of hospitalization. The last image displays a KUB X-ray film of the patient.

On the first day of hospitalization, levofloxacin at 500 mg/day was prescribed along with aggressive fluid resuscitation. However, by the second day, the patient’s condition had deteriorated and he was in a state of shock with a blood pressure of 80/50 mmHg. As a result, the decision was made to transfer the patient to the intensive care unit. Due to the presence of symptoms such as fever, jaundice, and acute renal failure, there was a suspicion of leptospirosis. Due to these critical conditions and some stomach upset, the antibiotic was changed to ceftriaxone at 2 g/day, and this treatment approach was continued throughout the patient’s hospitalization. The patient’s renal function parameters were notably high, and he experienced oliguria with a daily urine output of only 150 cc. To address this, renal replacement therapy was initiated and a Hickman catheter was inserted to facilitate the process. Following three hemodialysis procedures, urine output was normalized and his renal function was improved. The creatinine level was reduced to 1.3 mg/dL on day eight of hospitalization. However, hyperbilirubinemia, anemia, and thrombocytopenia worsened. Various serologic tests for infectious diseases were conducted, and the results were within normal limits for influenza A and B, HIV, ANA, hepatitis A and B, EB virus, CMV virus, and dengue NSI Ag. Because of suspicion of leptospirosis, serum from the patient at day three was sent for serology testing to the Centers for Disease Control (CDC). On the third day, the patient’s oxygenation worsened, and a new finding of bilateral infiltrates was observed on the chest x-ray (Figure 4). 

### 3.3. Respiratory Complications and Multifaceted Treatment Outcome of Leptospirosis

Fine rales were detected over both lung fields. The arterial blood gas results on 2 L of oxygen showed a pH of 7.44, PCO_2_ (partial pressure of carbon dioxide) of 35 mmHg, and PO_2_ (partial pressure of oxygen) of 58 mmHg, with oxygen saturation of 91%. Additionally, the serum level of bicarbonate is 23.8 mmol/L. To relieve dyspnea symptoms, the patient was provided with noninvasive positive pressure ventilation (BiPAP). However, at midnight of day 4, the patient experienced progressive dyspnea and irritation. The arterial blood gas analysis on 15 L of oxygen revealed a pH of 7.41, PCO_2_ of 43 mmHg, PO_2_ of 51 mmHg, oxygen saturation of 86%, and bicarbonate level of 27.3 mmol/L. In light of acute respiratory failure, endotracheal intubation was performed and the patient was transitioned to mechanical ventilation in pressure control ventilation (PCV) mode. The ventilator support was discontinued on the tenth hospital day. The color of the patient’s sputum, ranging from light to dark red, raised suspicion of pulmonary hemorrhage. In response to the patient’s worsening condition, which included acute renal failure, hyperbilirubinemia, and respiratory failure, a dose of methylprednisone (125 mg/Q6H) was initiated on the third day of hospitalization. After receiving treatments with renal replacement therapy, antibiotics, and respiratory support the patient’s overall condition improved significantly. During hospitalization, the urine output was normalized on day five after three hemodialysis procedures. The serum level of creatinine was 1.3 mg/dL on day eight of hospitalization, indicating a recovery in renal function. Consequently, we did not deem it necessary to apply further renal replacement therapy until the patient’s discharge. The patient was ultimately discharged with the normalization of renal and liver function parameters on the sixteenth hospital day. The diagnosis of leptospirosis was confirmed based on the results of a microscopic agglutination test (MAT), by the fourfold rise in antibody titers between acute and convalescent sera, from the CDC. Following discharge, the patient received a 5-day course of doxymycin with 100 mg every 12 h. Subsequently, the patient was monitored at our hospital outpatient clinic and exhibited overall positive progress. However, there was an isolated gout attack affecting the right ankle joint, for which appropriate medication was administered to address the issue.

## 4. Discussion

Leptospirosis is a global and potentially deadly zoonotic disease caused by pathogenic spirochetes belonging to the genus *Leptospira*. The organism has many known reservoirs, including small mammals, large herbivores, poikilothermic animals, and amphibians [3,9]. However, rodents remain the most significant reservoirs [10,11]. There was a rare reported case of severe leptospirosis presenting with cortical subarachnoid hemorrhage after bat exposure [12]. Incidental infections in humans are often linked to occupational, recreational, or avocational activities. Agricultural workers, such as those working in rice fields, banana farms, taro farms, sugar cane, and pineapple fields, are at a particularly high risk for contracting leptospirosis [13]. These occupations frequently involve exposure of skin wounds to soil and water that may be contaminated with the urine of rodents and wild or domestic animals, especially in tropical and subtropical regions. Another mode of transmission is through the ingestion of contaminated water or contact with it on mucous membranes. For instance, in a tropical region of Queensland, Australia, banana workers accounted for two-thirds of reported leptospirosis cases [14]. Some previous reports have suggested that dissemination of *Leptospira* in large animals may be attributed to sexual transmission. An intriguing study in mice infected with *Leptospira* demonstrated that live culturable *Leptospira* may be present in the testes during the acute phase (not at 15 days post-infection). This finding raises the possibility of mammal-to-mammal venereal transmission [15]. The mechanisms of *Leptospira* pathogenesis are still elusive. There is ongoing debate about the virulence of bacterial pathogens. The virulence of bacterial pathogens relies on their invasiveness and production of toxins. Exotoxins are not closely related to pathogenic *Leptospira* species. Various studies have shown that the *colA* gene product (a collagenase), high-temperature protein G, and some genes encoding proteins are closely related to virulence [16,17,18]. Based on the patient’s exposure history, it is likely that the patient became infected due to consuming contaminated underground water. In many urban areas, the routes of infection for patients are different from those in tropical and subtropical regions. According to the Taiwan CDC’s reports, there are approximately 27 to 203 cases of leptospirosis per year, with the peak occurring in the summer. The most common serotype is *L. shermani* (*Leptospira santarosai serovar Shermani*), which can infect both wild and domestic animals, with rodents being the primary reservoirs. The diagnosis of leptospirosis should not be overlooked in patients who present with an acute febrile illness, jaundice, and acute kidney injury.

Leptospirosis presents in two phases: the septicemic phase and the immune phase. In the septicemic phase (also known as the leptospiremic phase), bacteria can be cultured from blood, cerebrospinal fluid (CSF), and other tissues, but not from urine [19]. Common symptoms include flu-like illness with a high fever, myalgias, headache, and conjunctival suffusion. During the initial stage, patients typically experience a sudden onset of chills, fever, loss of appetite, nausea, vomiting, headache, and myalgias (usually affecting the paraspinal, abdominal, and calf muscles) [20]. It is important to consider other febrile illnesses such as influenza, dengue fever, malaria, or others during this phase. The immune phase (also called the leptospiruric phase) follows, characterized by the emergence of circulating antibodies and the presence of bacteria (spirochetes) in the urine [21]. Patients in this phase usually fall ill 7 to 12 days after exposure to leptospires, but this duration can vary from 2 to 20 days or longer [22]. The incubation time in leptospirosis is highly variable and may range from 6 to 29 days, as seen in the cases of 52 athletes who contracted leptospirosis after participating in the Springfield Triathlon. In this phase, an elevation of serum immunoglobulin M (IgM) can be observed, and bacteria can be cultured from the urine. Most research on leptospiral antigens has focused on lipopolysaccharide (LPS), with its predominant immunoglobulin M (IgM) antibody responses, but there is significant antigenic variability in leptospiral LPS. Guerreiro et al. [23] identified seven proteins (p76, p62, p48, p45, p41, p37, and p32) as targets in the humoral immune response during leptospiral infection. In the advanced stages of the disease, severe leptospirosis can lead to acute kidney failure, hepatic damage, pulmonary involvement, and meningitis or encephalitis. Weil’s disease, characterized by distinctive jaundice and acute renal failure, was first described in 1886 [21]. Aseptic meningitis may present with a range of symptoms, from none to disorientation or obtundation. The incidence of aseptic meningitis can affect up to 80% of patients [15]. Renal symptoms, such as hematuria, pyuria, azotemia, and acute renal failure, may occur. It has been reported that pulmonary involvement in human leptospirosis occurs in approximately 20% to 70% of cases, and its incidence is increasing [7]. Radiographically, cases of pulmonary involvement may show small nodular densities, a ground glass appearance, and areas of consolidation on imaging studies. An autopsy study found that pulmonary hemorrhage remained a primary cause of death in leptospirosis cases [24]. Alveolar infiltration on chest X-rays, along with dyspnea, has been observed as a poor prognostic indicator in severe leptospirosis. Pulmonary manifestations may range from cough, chest pain, dyspnea, and hemoptysis to life-threatening acute respiratory distress syndrome (ARDS). In a cohort study of 26 Spanish patients, 17 of them experienced respiratory problems (65%), and three patients died after an ARDS event occurred [25]. In the present case, the patient, who initially suffered from dyspnea with bilateral lung infiltration on chest X-rays, rapidly progressed to acute respiratory failure and required ventilatory support.

Weil’s disease is the most severe clinical presentation of leptospirosis, in which the pathogen spreads widely through the bloodstream. Patients progress to multisystem organ failure with clinical symptoms such as high fever (>40 °C), circulatory system collapse, hepatocellular damage, renal failure, pulmonary dysfunction, central nervous system changes, and coagulation abnormalities, and clinical courses can vary. In general, the disease mortality rate may reach up to 22%, but this also depends on the quality of local healthcare facilities, environmental hygiene, and governmental health policies [26]. When fulminant Weil’s disease presents with cardiovascular collapse and pulmonary hemorrhagic pneumonitis, the mortality rate may be as high as 50% [27,28]. Several case reports highlight instances of severe leptospirosis leading to multiorgan failure. A comparative analysis of these reports reveals that leptospirosis typically presents with initial nonspecific symptoms, including fever, headache, general fatigue, weakness, nausea, and myalgia. In certain cases, the disease progresses rapidly, leading to renal, liver, and pulmonary involvement. Respiratory failure, necessitating intubation, may occur, and it is often associated with alveolar lesions predominantly located peripherally in chest X-rays. Thrombocytopenia, hyperbilirubinemia, and impaired renal function emerge as common features in these cases. Notably, in one documented case, Methylprednisolone at a dosage of 1 g once daily was utilized to manage the critical condition [29,30]. Another related phenomenon is a cytokine storm. Its pathogenesis involves *Leptospira* outer membrane proteins (OMPs) activating nuclear transcription factor kappa B (NF-kB) and mitogen-activated protein kinases (MAPK) via a Toll-like receptor (TLR)-dependent pathway. This process can result in tubular injury and inflammation. The induced chemokines and cytokines elicit systemic inflammation. *Leptospira* OMPs may also activate the transforming growth factor-β (TGF-β)/Smad-associated fibrosis pathway, leading to extracellular matrix accumulation [31]. Patients with Weil’s disease may present with a cytokine storm characterized by high levels of IL-6, IL-10, and tumor necrosis factor-alpha (TNF-α) [32]. TNF-α may be considered an important predictor of the severity of leptospiral infection. Compared with patients without serious complications, significantly higher levels of circulating TNF-α may be observed in severe leptospiral infection with kidney, liver, and lung involvement [33]. The use of steroid treatment for Weil’s disease is a subject of controversy. In this case, we did not initially prescribe steroids for this disease. However, when the patient’s condition became critical with circulatory collapse, liver damage resulting in high bilirubin levels, acute renal failure necessitating hemodialysis therapy, and respiratory failure requiring ventilator support, steroids were administered to alleviate the critical conditions, in addition to the use of appropriate antibiotics. The typical clinical manifestations of leptospirosis are diverse, ranging from mild influenza-like illness to severe life-threatening conditions such as Weil’s disease, pulmonary hemorrhage, and central nervous system involvement. Around 5–10% of all leptospirosis cases progress to severe forms of the disease with a mortality rate of 5–15% [20]. A study from a suburban area in Malaysia analyzed 42 patients with a confirmed diagnosis of leptospirosis. They identified some predictors with a high mortality rate, including age ≥ 70, breathlessness, positive lung findings, oliguria, use of vasopressors, administration of steroids, the need for ventilator support, and blood transfusion [3]. 

Interestingly, the kidney is the primary target in leptospirosis, with clinical presentations ranging from abnormal urinary sediment to acute renal failure requiring renal replacement therapy. As in this case, the urine routine test showed abnormal sediment with the presence of proteinuria, hematuria, and pyuria. The incidence of acute renal failure varies widely, ranging from 40 to 60% in severe leptospirosis [34]. In the reservoir host, pathogenic leptospires can spread hematogenously, invade the kidneys, and colonize the tubular lumen. Immunohistochemistry can reveal the presence of large numbers of pathogenic leptospires in the brush border of the proximal tubular epithelium, leading to subsequent tubulointerstitial nephritis and mononuclear cellular infiltration [35]. A leptospiral glycolipoprotein, a component of the outer membrane, is responsible for host-pathogen interactions and eliciting immune responses [36]. In a study conducted by Yang et al. [37], they applied leptospiral OMP extract to cultured mouse renal tubular epithelial cells to elucidate the mechanism of tubulointerstitial injury caused by leptospiral infection. They found that leptospiral OMP can activate the expression of NF-kB activator protein-1 and some downstream genes in medullary thick limb cells [35].

**Figure 5:**
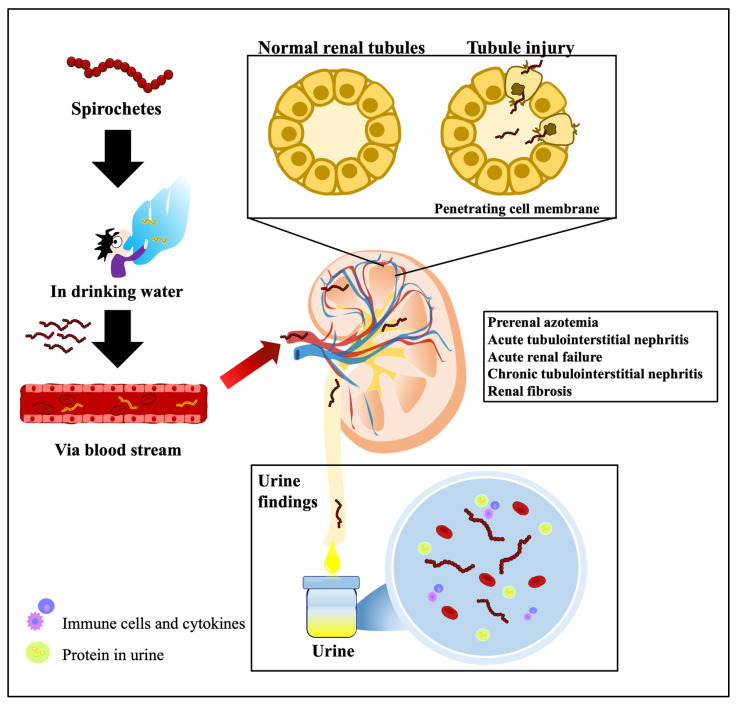
The linkage between the spirochete and kidney impairment.

In clinical practice, the main renal lesion involved in leptospirosis is acute interstitial nephritis with or without acute tubular necrosis but not glomerular changes. When acute interstitial nephritis occurs, an ultrasound may reveal enlarged kidneys with increased echogenicity. In this patient, mild enlargement of the left kidney (right kidney size 11.07 cm, left kidney size 12.56 cm) and increased echogenicity in both kidneys were observed. The clinical presentations of acute renal failure are characterized by initial azotemia, oliguria/anuria, and uremia, commonly noticed in the second week of the disease along with jaundice [38]. However, in this case, acute renal failure was observed on the second day of hospitalization, and the patient required maintenance dialysis due to oliguria, azotemia, and hypotension. The primary causes of acute renal failure in leptospirosis are tubulointerstitial nephritis. The development of acute tubular necrosis is influenced by hemodynamic changes resulting from severe hypotension, subsequent immune responses, and direct nephrotoxicity (Figure 5). Early dialysis treatment has been shown to be beneficial for renal function recovery and disease prognosis. During the course of the disease, proximal tubular lesions enhance distal sodium delivery, leading to potassium wasting in the distal tubule [39]. As a result, some cases may present with hypokalemia and hyponatremia. In this particular case, hypokalemia and hyponatremia (sodium 132 mmol/L and potassium 3.5 mmol/L on the first day of admission) co-existed as clinical signs of tubular dysfunction in leptospirosis. Malfunctioning of the medullary thick ascending Na^+^-K^+^-Cl-cotransporter (NKCC2) has also been observed in *Leptospira santarosai serovar Shermani*-infected patients with hypokalemia [32].

A cytokine storm is triggered by an uncontrolled immune response. It is a deadly systemic inflammatory syndrome that involves the hyperactivation of immune cells and a rise in circulating cytokines. This phenomenon can be caused by various pathogens, cancers, autoimmune diseases, and certain therapies [40]. In patients with severe leptospirosis, elevated levels of IL-6, IL-10, and TNF-α have been observed during a cytokine storm [3]. In leptospirosis, cytokine storm is considered the primary factor contributing to Weil’s disease, which leads to a rapidly progressive ARDS and multiple-organ failure. The potent invasive ability of *Leptospira* species may play an important role in rapid systemic infection, leading to a cytokine storm. A recent study highlighted that the endocytosis processes in human macrophages and human vascular endothelial cells during leptospirosis differ significantly due to diverse mechanisms. Particularly, the swift transcytosis through vascular endothelial cells mediated by ITGB1/CAV-1/PI3K-signaling may elucidate certain mechanisms underlying severe systemic infection and the subsequent cytokine storm [41]. Concerning immunity, the interaction between innate and adaptive immune responses is crucial, with dendritic cells (DCs) orchestrating these responses. A study has provided novel insights, indicating that pathogenic leptospires can impair the maturation of monocyte-derived dendritic cells (MoDCs), consequently compromising the efficacy of CD4+ T cell proliferation. In this situation, pathogenic leptospires can modulate the host immune response, resulting in systemic dissemination [42]. The above evidence partially explains why pathogenic leptospires easily cause systemic dissemination and induce dysregulated immune responses within the host. Regarding the pathogenesis of cytokine storms, it is extremely complex, requiring further efforts for a clearer understanding. To address this, high doses of steroids have been suggested as a means to mitigate the effects of cytokine storm in severe leptospirosis, resulting in improved outcomes [43,44]. 

The limitations of this study include: (1) Leptospirosis typically spreads in high rainfall tropical regions, rural and urban slum communities with inadequate sanitation and poor housing, as well as travel areas. In our case, the patient lived on the outskirts of an urban setting with some stray animals. It is important to note that leptospirosis is only sporadic and not annually endemic in Taiwan. (2) Diagnostic capacity in Taiwan CDC is limited. We are unable to perform blood culture and PCR for pathogenic *Leptospira* in the septicemic phase, which could enhance the sensitivity and specificity of diagnosis. (3) The final diagnosis in this case relied on the MAT method. MAT is a complex test that requires control, skilled performance, and interpretation by highly experienced personnel. Despite these limitations, the patient exhibited typical prodromal symptoms and clinical presentations, and we endeavored to administer accurate treatments. Additionally, CDC support for our diagnosis was obtained through a positive MAT report.

## 5. Conclusions

In our case, the patient was admitted with a rapidly deteriorating clinical condition, characterized by high fever and severe hypotension. Despite unavailable access for checking serum levels of IL-6, IL-10, and TNF-α in in our health insurance system, a potentially fatal cytokine storm was highly suspected. Based on our knowledge, we decided to use a nonspecific immunosuppressive agent (methylprednisolone) to counteract the cytokine storm in the early stage. Despite the patient experiencing acute respiratory and renal failure, there was noticeable improvement in the following days with better results. Nevertheless, we must be cautious about the potential risk of future infections due to the use of immunosuppressive agents. Further clinical trials may be necessary to investigate this matter further.

## Figures and Tables

**Table 1:** Laboratory test results and vital sign changes during hospitalization.

Days of Hospitalization	1	2	3	4	5	6	7	8	9	10	11	15
Temperature (°C)	39	38.4	37.5	37.5	38.7	37.6	38	37.6	39	37.5	37.5	37
Blood pressure (mmHg)	87/51	80/44	88/52	100/64	112/58	89/65	106/96	106/83	107/74	115/68	113/73	113/78
Dialysis			▲	▲	▲							
Endo. Tube				★	★	★	★	★	★	★		
WBC (10^3^/μL) (3.9~10.6)	13,760	13,310	17,160	16,100	26,010	35,700		24,770		17,560		11,050
Neutrophils (%) (40–75)	80	91	84	78	70	73		71		69		70
Lymphocytes (%) (20–45)	11	5	10	5	8	9		12		25		24
Monocyte (%) (2–10)	1	3	3	2	4	8		3		5		4
Eosinophil (%) (0.2–6)	0	0	0	0	0	0		0		0		0
Band (5) (0–5)	0	0	3	0	5	5		1		0		0
Hb (10^6^/μL) (13.5–17.5)	10.8	5	6.1	7.5	7.6	9.3		9.3		10		10.5
Platelet (10^3^/μL) (150–400)	23	20	45	130	196	179		219		374		439
ESR (mm) (0–10)		115					66					
Blood Gas Analysis			Nasal Cannula 3 L	BiPAP 15 L/min		Ventilator O_2_ 70%				Ventilator O_2_ 30%		
PH (7.35–7.45)		7.394	7.44	7.41		7.49				7.49		
PCO_2_ (mmHg) (35–45)		32.2	35	43		43				38		
PO_2_ (mmHg) (80–100)		66.9	58	51		158				109		
HCO_3_ (mmol/L)		19.2	23.8	27.6		32.6				29		
O_2_ SAT(%) (95–100)		92.6	NA	86		100				99		
BUN (mg/dL) (7~25)	25		63	55				59		31		
Cr (mg/dL) (0.7–1.3)	2.2	3.9	5.5	3.7				1.3		0.8		0.7
GFR (ml/min/1.73 m^2^) (>90)	35.78	18.48	12.43	19.64				65.66		114.99		134.14
Na (mmol/L) (136–146)	131	132	132					155		159		138
K (mmol/L) (3.5–5.1)	3.5		3.8			3	2.6	2.7		3.4		4.4
Ca (mg/dL) (8.4–10.2)	8.2											
AST (mmol/L) (13–39)		375	402			88	5.7	56		53		
ALT (U/L) (7–25)	39	54	66			48	36	38		46		
LDH (U/L) (140–270)	314	476										
CK (U/L) (30–223)	8047	14,947										
Bilirubin D/T (mg/dL) (0.03–0.18, 0.3–1.0)	1.73/3.8	2.66/4.7		9.19/14.1		2.98/4.8	1.13/2.3	0.92/2.0		0.72/1.7		
Glucose (mg/dL) (70–100)	109					107		112				
CRP (mg/dL) (<0.3)		21.94					9.15	5.53				
Albumin (g/dL) (3.5–5.7)				2.8						3		

▲: one course for intermittent hemodialysis (IHD). ★: intubation with a ventilator.

## Data Availability

The original contributions presented in the study are included in the article, further inquiries can be directed to the corresponding author.
